# What is the effectiveness and safety of mirtazapine versus escitalopram in alleviating cancer-associated poly-symptomatology (the MIR-P study)? A mixed-method randomized controlled trial protocol

**DOI:** 10.1186/s12904-022-00976-7

**Published:** 2022-05-23

**Authors:** Guillaume Economos, Marine Alexandre, Elise Perceau-Chambard, Laurent Villeneuve, Fabien Subtil, Julie Haesebaert, Olivier Glehen

**Affiliations:** 1 EA 3738 - CICLy – Centre d’Innovation en Cancérologie de Lyon, UFR Faculté de Médecin Lyon-Sud-Charles Mérieux BP1, Université Claude Bernard Lyon 1, 165, Chemin du Grand Revoyet, 69921 Oullins Cedex, France; 2 Palliative Care Center, Hospices Civils de Lyon, 165, Chemin du Grand Revoyet, 69495 Pierre-Bénite, France; 3 Public Health, Research and Clinical Epidemiology, Hospices Civils de Lyon, 165, Chemin du Grand Revoyet, 69495 Pierre-Bénite, France; 4 Surgical Oncology, Hospices Civils de Lyon, 165, Chemin du Grand Revoyet, 69495 Pierre-Bénite, France

**Keywords:** Signs and Symptoms, Neoplasms, Advanced cancer, Palliative Care, Antidepressive Agents, Polysymptomatology, Cancer pain

## Abstract

**Background:**

Advanced cancer patients often experience multiple symptoms at a same time. This might lead to polypharmacy and increase adverse events representing major threats to the quality of health care, especially in palliative care situations. Mirtazapine, an antidepressant agent, has been suggested as a potential relevant drug to alleviate multiple cancer-related symptoms at a same time.

Therefore, the present study aims to assess the effectiveness of mirtazapine in alleviating multiple symptoms at a same time in advanced cancer patients suffering from a major depressive episode compared to a group receiving escitalopram, another antidepressant agent.

**Methods:**

Multicentre, prospective, randomized, controlled trial in 12 palliative care services in France.

The study will be based on a mixed-method methodology using parallel groups, of oral mirtazapine compared with oral escitalopram, with a 56 day follow-up. The primary outcome will be an improvement of the Global health Status (issued from the EORTC-QLQ-C30) on day 56. 418 participants will be clinically followed-up on day 7 and 56 and will have a telephonic assessment on days 14 and 28. A sub-sample of participants will be invited to take part in semi-structured qualitative interviews at baseline and day 56. For the qualitative part, purposeful sampling will be used.

**Discussion:**

This study will provide evidence for the pharmaceutics management of poly-symptomatology in advanced cancer patients. This could lead to important changes in the management of those patients by using a single molecule to alleviate multiple symptoms at a same time, potentially improving medication adherence, symptoms’ control, and reducing the risk of medications adverse events.

**Trial registration:**

Trial registration: NCT04763135. Registered 18 March 2021.

## Background

Despite continuous progress in cancer specific treatments, cancer remains the first leading cause of death in European countries. Therefore, the scientific community has an increasing interest in improving cancer patient’s quality of life [1, 2].

Cancer patients are highly burdened by symptoms with variability depending on cancer site, stage, disease trajectory, and cancer treatments [3, 4]. This burden worsens with the disease progression. For this reason, advanced cancer patients very often experience symptoms altering their quality of life, while the aim, at this stage, is to improve it. Indeed, more than half of advanced cancer patients experience fatigue, pain, lack of energy, tiredness or lack of appetite. Unfortunately, as patients often experience multiple symptoms at the same time, they might need multiple drugs, resulting in higher risk of adverse event or drug interactions [5, 6]. A potential solution to avoid such an issue might be to use a single drug to target multiple symptoms at the same time. Additionally, very few treatments are available for some symptoms, for instance, no treatment is currently licensed in Europe for breathlessness and a very few are available for lack of appetite or loss of weight.

Mirtazapine is a licensed anti-depressive treatment, advocated to be an eligible drug in treating multiple cancer related symptoms [7, 8]. Indeed, mirtazapine has an α-2 adrenergic activity which increases noradrenergic and serotoninergic central neurotransmitters, leading to its short-term effect on acute depressive disorders [9]. Besides this α-2 adrenergic activity, mirtazapine also links to 5HT3 receptors and H1 receptors. This activity on various central and periphery receptors might have various effects on appetite, sleep disorders, pruritus, emesis, breathlessness and other multiple symptoms. Numerous recent studies gave additional clinical argument to support mirtazapine as an effective treatment for sleep disorders [10], breathlessness [11], and lack of appetite in advanced cancer patients [12].

Unfortunately, our previously published review highlighted that, despite encouraging results in various symptoms, evidence for the effectiveness of mirtazapine in improving quality of life in cancer patients experiencing poly-symptomatology is still lacking of robust randomized controlled trials [13].

Because health-related quality of life is a highly associated with the level of burden driven by symptoms; our research aims to assess the effectiveness of mirtazapine in improving health-related quality-of-life (HRQoL) in advanced cancer patients experiencing poly-symptomatology.

### Hypothesis

We hypothesize that oral mirtazapine is superior to another antidepressant drug (oral escitalopram) in improving health-related quality of life of poly-symptomatic advanced cancer patients suffering from depression.

### Objectives

The primary objective will be to assess the effectiveness of oral mirtazapine in improving HRQoL in poly-symptomatic advanced cancer patients suffering from depression after 56 days of treatment, compared with oral escitalopram.

The secondary objectives are:

i- To assess the effectiveness of oral mirtazapine in poly-symptomatic advanced cancer patients suffering from depression compared with escitalopram on the following outcomes:

- The experience of poly-symptomatology, poly-medication, and taking antidepressant agents and explore the dynamic of this experience between inclusion and 56 days.
- The improvement of the following symptom intensity: depressive mood, pain, nausea, lack of appetite, vomiting, breathlessness, sleep disorders and anxiety at 28 and 56 days.
- The improvement of the different functional capacities at 56 days.
- The mitigation of the loss of weight at 28 and 56 days.
- The use of medications to treat associated symptoms at 28 and 56 days.

ii- To assess the safety of oral mirtazapine in poly-symptomatic advanced cancer patients suffering from depression compared with escitalopram from 7 days.

iii- To assess the compliance to the treatments.

## Methods

**Fig. 1:**
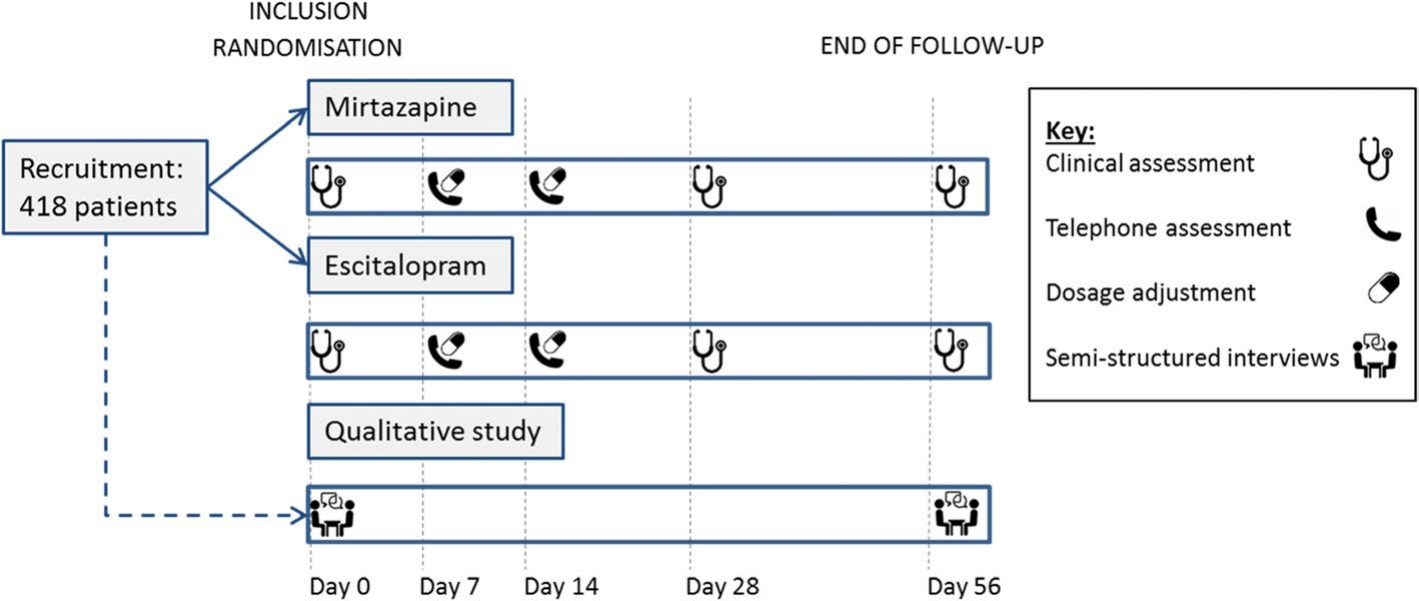
Overview of the study

The trial will be a multicenter, prospective, randomized, open-labelled, controlled trial based on a mixed-method methodology using parallel groups, of oral mirtazapine (intervention) compared with oral escitalopram (control), with a 56-day follow-up. Semi-structures interviews will be performed on a purposive sample for qualitative outcomes. The 418 participants will be followed-up on day 7, 14, 28 and 56 for a 56-day period. A sub-group of participants will be invited to take part in qualitative interviews at baseline and day 56. Recruitment of participants for the qualitative part will be based on a purposive sampling. An overview of the study (Fig. 1) and an overview of the study events (Table 1) are provided below.

**Table 1:** Overview of the study events for each participant during the follow-up period

***STEPS***	***Screening***	***Inclusion*** ***Randomisation***	***V2*** ***Telephone call***	***V3*** ***Telephone call***	***V4*** ***Clinical assessment***^c^	***V5*** ***Clinical assessment***^c^
***Time***	***J-14 à J-2***	***J0***	***J7*** ** ± *1 j***	***J14*** ** ± *1 j***	***J28* ± *3 j***	***J56* ± *3 j***
***Actions***						
***Inclusion and non-inclusion criteria***	*X*	*X*				
***ECG et postural hypotension test***		*X*				
***Study information delivery***	*X*	*X*				
***Informed consent***		*X*				
***MADRS***	*X*	*(X)*^b^			*X*	*X*
***HADS-D***	*X*	*(X)*^b^			*X*	*X*
***QLQ-C30***		*X*			*X*	*X*
***ESAS12-F***		*X*			*X*	*X*
***ASEC***		*X*	*X*	*X*	*X*	*X*
***Factor 1 of the MARS***					*X*	*X*
***Clinical assessment: weight, blood pressure, Performans Status***		*X*			*X*	*X*
***Treatment list***		*X*			*X*	*X*
***Sociodemographic data***^a^		*X*				
***Randomisation***		*X*				
***Treatment doses adaptation***		*X*	*(X)*	*X*	*X*	*X*
***Adhesion to treatment assessment (pill count)***					*X*	*X*
***Semi-structured interviews***		*X*				*X*
***Side effects***		*X*	*X*	*X*	*X*	*X*

^a^***Socio-demographics:**** date of birth, marital status, number of children, declared level of autonomy/dependence, professional position, way of life, kind of home support, cancer type, stage of the cancer, prior and current cancer treatments, personal background of psychotropic drugs use, the existence of a psychological or nutritional follow-up (and frequency of it)*

^b^***HADS-D/MADRS:**** if not available at the time of screening or older than 7 days old*

^c^***Clinical assessment:**** might be performed using teleconsultation if needed*

### Population

Our study has interest in the population of adult patients suffering from an advanced cancer, who are diagnosed from a depressive syndrome and are experiencing at least another symptom.

### Sample Size

Based on an expected standard deviation of 26 in our primary outcome (The Global health Status), and considering a correlation of 0.5 between inclusion and day 56 (corresponding in a standard deviation of the variation of the GHS score of 26 between baseline and day 56), and expecting a moderately clinically significant difference of 8 points between groups, it will be necessary to include 418 participants (bilateral test, alpha risk of 0.05, power of 0.8, attrition 0.2).

Considering the risk of lost to follow-up due to our population global condition, we considered an attrition rate of 20% when calculate the sample size.

### Inclusion and non-inclusion criteria

Inclusion and non-inclusion criteria are summarized in Table 2.

**Table 2:** Inclusion and non-inclusion criteria

Inclusion criteria	Non-inclusion criteria
Being over 18 years old	Being treated with an antidepressant agent during the four weeks before inclusion
Suffering from an advanced cancer	Having had a hypersensivity event to mirtazapine, escitalopram of any excipient
Having a clinically estimated life expectancy over 3 months	Having had a prior inefficient treatment with mirtazapine or escitalopram
	Having postural hypotension or arterial systolic hypotension inferior to 90 mmHg measured following the guidelines of the European Society of Cardiology
Being diagnosed from having a depressive syndrome by a Hospital Anxiety and Depression Scale-D over 11	Having a QT interval over 420 ms
	Having uncontrolled heart rhythm disorder or uncontrolled conduction disorder
Being in need of an antidepressant treatment	Having had or having bipolar disorder
Suffering from at least one under-controlled symptom (defined as a score over 3 on the Edmonton Symptom Assessment Scale) among: pain, nausea, vomiting, breathlessness, lack of appetite, sleep disorders, or anxiety	Having uncontrolled seizure or epilepsy (relative non-inclusion criteria needing a neurology specialist opinion)
	Having or having a history of closed-angle glaucoma
Having or not a cancer treatment	Having bone marrow aplasia
Being able to understand the information related to the study, and to sign informed consent	Practicing breastfeeding or being pregnant
Having agreed to take part in the study	Women of childbearing age with no contraception method
	Having a treatment with: - Monoamine oxidase inhibitors (Selegiline, Moclobemide, Isocarboxazid, Nialamide, Phenelzine, Tranylcypromine, Iproniazid, Iproclozide, Toloxatone, Linezolid, Safinamide, Rasagiline) - One of the following antiarrhythmic drugs: Flecainde, Propafenone, any class IA and III antiarrhythmic drug (amiodarone, disopyramide, hydroquinidine, quinidine, procainamide, sparteine, ajmaline, prajmaline, lorajmine, bretylium tosilate, bunaftine, dofetilide, ibutilide, tedisamil, dronedarone) - Antipsychotic drugs (phenothyazine antipsychotics, pimozide, haloperidol) - Linezolid, sparfloxacin, moxifloxacin, macrolids (IV erythromycin, josamycin, clarithromycin, telithromycin), pentamidin, halofantrine, HIV protease inhibitors (ritonavir, nelfinavir, amprenavir, indinavir), azolic antifungal agents (ketoconazole, itraconazole, miconazole, fluconazole, voriconazole) - Mizolastine and Cimetidine - Ticlopidine - Metoprolol - Methadone - Ketamine - Triptan drugs - Dapoxetine - St. John's wort - Antidepressant drug - Any other medication known to cause prolonged QT intervals
Being able to fill Patient Reported Outcomes questionnaires	Having genetic galactose intolerance or glucose-galactose malabsorption
	Having one of the following electrolyte disorders not corrected at the time of inclusion: hyponatremia, hyperkalemia, hypokalemia, hypermagnesemia, and hypomagnesemia
Being available to be called on days 7 and 14	Having end-stage renal disease with a creatinine clearance inferior to 15 ml/min calculated using the Cockroft’s formula
	Having hepatic failure
Having a social security affiliation	Having legal incapacity

There is no unanimous definition of advanced cancer. Scientific societies mainly agree to define advanced cancer as conditions that cannot be cured in the current state or science (Canadian cancer society, ESMO, [14]. However, they disagree with including the metastatic status (ESMO, [14]), impossibility to perform surgery (ESMO), or lack of response to first line treatments (CCS).

Based on these various definitions, in our study, we will rely on the following definition: “cancers that cannot be cured, and that, in the case of metastatic cancer, have usually spread from where they started to other parts of the body.”, [15] in addition to “cancers that progress after a second line specific treatment or primary location of cancer that is unresectable”.

Patients will be considered to have a depressive syndrome if their Hospital Anxiety and Depression Scale-D score rates over 11(37), and if the clinical assessment supported by the results from the Montgomery and Åsberg Depression Ratting Scale (38), confirm the diagnosis and the need for an antidepressant treatment.

### Recruitment sites

Eleven French centers will handle recruitment. All centers have specialized palliative care services on site, including a palliative care unit. All of them take care of advanced cancer patients on a regular basis. The centers are seven University Hospitals, two Comprehensive Cancer Centers, one Public Hospital and one non-profit private hospital.

For practical justifications, the recruitment for the qualitative part of the study will be made only in one center. Centers are located in various French regions.

### Study procedure

#### Recruitment and inclusion procedure

A screening for potential participants will be made by clinical teams in oncology or palliative care, during consultations, day hospital or conventional hospitalization. The principal investigator will be informed of every potential participant. He will verify inclusion and non-inclusion criteria. If the potential participant fulfils all requirements, the principal investigator will meet the patient to give information about the study (and about the qualitative part of it if needed).

Then the participant will be called at least 48 h after having received the oral and written information. They will be asked if they want to part to the study. If positive, an appointment for inclusion, randomization and baseline assessment will be booked. At inclusion, participants will sign an informed consent to participate. For those who will take part in the qualitative part, they will receive separate information and sign a specific consent form.

At inclusion, participants will be clinically examined with a postural hypotension test and an ECG if no prior ECG is available. If the participant does not have any non-inclusion criteria, he will be enrolled in the study and receive an anonymity code generated by the ENNOV Clinical software. Socio-demographic data will be collected. The participant will have to fulfil the EORTC QLQ-C30, Hospital Anxiety and Depression Scale –Depression (HADS-D), Edmonton Symptom Assessment Scale 12 French (ESAS-12F), and Antidepressive Side-Effect Checklist (ASEC) questionnaires. The principal investigator will fulfil the MADRS questionnaire and assess the patient’s Performance Status. If needed, the principal investigator will be able to ask for a psychiatric opinion.

#### Randomization

On the day on inclusion, participants will be randomly assigned to one of the two arms of the study by the ENNOV Clinical software. We will use the minimization method. Due to the important influence on quality of life, the Performance status, and the number of symptoms for which intensity is over 3 will be considered during randomization. The inclusion centre will also be considered.

#### Intervention and comparator

Mirtazapine will be compared to another antidepressant drug to avoid confusion bias in improving health-related quality of life due to the lone antidepressant effect of mirtazapine. Escitalopram has been chosen as a comparator because it is, like mirtazapine, effective in treating depression in advanced cancer [16], with few medication interactions [17], but no effect on poly-symptomatology is expected.

Both drugs will be used following the usual practice for depression. They will be introduced at a low dose regularly increase at day 7, 14 and 28 depending on the expected benefits from a higher dose and potential dose-dependent side effects. (Table 3).

**Table 3:** Doses escalation during the study follow up

		**Starting doses – Day 0**	**Day 7**	**Day 14**	**Day 28**
		Mirtazapine: 15 mg Escitalopram: 10 mg (or 5 mg for patients older than 65)			
Excessive drowsiness (CTCAE grade 1 or rated moderate or severe using the ASEC)			*For patients under 65 in the escitalopram arm: decrease to 5 mg*		
			*For every other patients: Stop the treatment*		
Any severe adverse effect or serious adverse event (CTCAE grade > 3 or rated severe using the l’ASEC)			*Stop the treatment*		
Moderate adverse event (CTCAE grade 2 or rated moderate using the ASEC)			Decrease the dosage (Or treatment stop if already at the lower dosage)		
Mild adverse event (CTCAE grade 1 or rated mild using the ASEC)			No dose adjustment		
No adverse event	Inadequate symptoms’ control (ESAS > 3)		No dose adjustment (or increase the dosage if escitalopram has been started at 5 mg)	Increase the dosage	
	Adequate symptoms’ control (ESAS ≤ 3)		No dose adjustment		

#### Follow-up procedure

Follow-up will be made on a regular basis during a 56-day period.

Patients will be called by a research assistant on days 7 and 14 to screen for side effects using the ASEC scale. If any adverse event is screened a clinical opinion will be sought for dose adaptation based on predefined criteria. On day 14, escalation of dose will be prescribed by the PI if symptoms are still under controlled (the corresponding ESAS12-F subscale rated over 3) and in the absence of limiting side effect evaluated using the ASEC and Common Terminology Criteria for Adverse Events V4.0.

Clinical assessment will occur on day 28 (more or less 3 days). This assessment is meant to be made physically; however, if the patients’ condition of public-health situation demands it, it will be performed using teleconsultation. During this consultation, the patient will fulfil the EORTC QLQ-C30, ESAS -12F, HADS-D, and ASEC questionnaires. The PI will weigh the participant, count the number of pills that remains to the patient, evaluate the Performance Status and fill the MARDS. Considering all available data (tolerance, side-effects and symptom control), the PI will make the decision regarding the escalation of treatment dose.

During the final evaluation, on day 56, the patient will fulfil the EORTC QLQ-C30, ESAS -12F, HADS-D, ASEC, and factor 1 of the Medication Adherence Rating Scale (MARS) questionnaires. The PI will weigh the participant, count the number of pills that remains to the patient, evaluate the Performance Status and fill the MADRS.

#### Outcomes assessment

The outcomes will mainly be assessed using patients reported outcome measurements (PROMs) to better reflect the patients’ experience associated with the evaluated treatment. As depressive self-assessment of depressive state might not capture dynamic changes in depressive mood, the depressive mood assessment will rely on a self-assessment, a hetero-assessment and a composite criterion.

The primary outcome will be difference in the health related quality of life, measured at baseline and day 56 using the Global Health Status (GHS) score derived from the EORTC-QLQ-C30 [18–20]. We will consider that a small difference in the GHS score will be between 4 and 8, and a medium difference will be over 8 between baseline and day 56 [21, 22].

The secondary outcomes will include the improvement in the patient’s dynamic experience between baseline and day 56 of the negative experience of symptoms, explored using semi-structured interviews focusing on the experience of symptoms, polymedication and taking antidepressant drugs.

The proportion of symptoms, for each patient, that have been reported as less intense using the ESAS-12F scale between baseline and day 56 if they were reported at baseline, among: depressive mood, pain, nausea, vomiting, loss of appetite, breathlessness, sleep disorders, and anxiety. A lesser degree of intensity will be considered from a 1 point difference [23].

The improvement in the intensity of each symptom using the ESAS-12F subscales between baseline, day 28 and day 56, if they were reported at baseline. A modification in the symptom’s intensity will be considered from a 1 point difference [23].

Any improvement in depressive mood will be assessed using the HADS-D scale and considering a modification in the patient’s mood from a 1.5 points difference between baseline, day 28 and day 56 [24]. We will also use a hetero evaluation scale, the Montgomery-Åsberg Depression Ratting Scale (MADRS) to compare depressive mood between baseline, day 28 and day 56. A difference over 1.9 points will be considered as significant [25]. A composite criterion of both scales’ improvement will also be assessed between baseline, day 28 and day 56 [24, 25].

The proportion of patients having an improvement in the different EORCT-QLQ-C30 functioning scales will be compared between baseline, day 28 and day 56. The following cut-offs will be used for defining significant changes: 3 points for the Cognitive Functioning, 2 points for the Physical functioning, and 6 points for the Emotional Functioning and Role functioning [26, 27]. Financial functioning will not be assessed as the French health care system does not fit with this evaluation (cancer care is free of charge to the patient).

Weight variations will be assessed between baseline, day 28 and day 56.

The use of painkillers will be assessed using the proportion of patients that had stable (± 20%) morphine oral equivalent between baseline, day 28 and day 56. For other symptomatic treatments, the proportion of patients that increased the doses (or had new symptomatic treatment) between baseline, day 28 and day 56 will be assessed.

The security of use will be assessed using the number of new adverse effects reported using the ASEC on day 14, 28 and 56 and compared with baseline. Adverse events will also be recorded and graded using the CTCAE V4.0. The ESAS12-F subscales for drowsiness, fatigue and constipation will be used to compare these symptoms between baseline, day 28 and day 56. A higher degree of intensity will be considered from a 1 point difference [23].

Finally, adherence to medication will be assed using the MARS and Proportion for Days Covered at day 56 [28].

### Statistics

The mean Global Health Status (GHS) in our population has been esteemed to 37.5 ± 10.4 [29] or 39 ± 26 [30]. Considering a standard deviation equal to 26 under the hypotheses that (i) the variance of the GHS score at inclusion and at day 56 are equal, and that (ii) the correlation between the score at day 0 and day 56 is equal to 0.5, and expecting a clinically significant change in the GHS of 8 points between day 0 and day 56, we calculated that 167 participants are needed in each group for an 80% study power. Considering the high risk of attrition in our population, we increased by 20% the number of participants needed, resulting in a total number of patients needed to 418 participants.

Analyses will be performed in intention to treat. The intention to treat population will be defined as all patients included in the study, regardless if they are assessed for the primary outcome or not. A modified intention to treat population will be defined as the intention to treat population without participants deceased before the end-point or for whom the primary outcome is missing.

Analyses will be performed using the SAS 9.4 software (SAS Institute Inc., Cary, NC, USA), considering an alpha-risk of 0.5.

### Qualitative part procedure

We will perform a qualitative comparative analysis of semi-structured interviews.

Only patients from the coordination center will be offered to take part in this sub-study.

### Legal and administrative considerations

The current protocol has been approved by the Committee for Persons Protection Sud Est I. On the 17/12/2020 with reference MEDAECPP-2020–10-00,020.

The study fulfilled all administrative requirements for clinical trials under the French law.

No data monitoring committee will set up. The sponsor will handle data monitoring twice a year for the time of the study and following the French legal requirements.

The study design complies with the Consolidated Standards of Reporting Trials (CONSORT) statement, and the current protocol complies with the Standard Protocol Items: Recommendations for Interventional Trials (SPIRIT) [31].

## Discussion

If mirtazapine is proven to be efficient in improving patients’ quality of life, independently of its antidepressant effect, in advanced cancer poly-symptomatic patients would constitute a major progress. Additionally, this study might highlight target symptoms that could be relieved using mirtazapine and potential target symptom clusters.

This could allow limiting polypharmacy in advanced cancer patients by offering a new therapeutic option well tolerated in this frail population. Additionally, this would mitigate the risk of adverse event and drugs interaction in this population often affected by polypharmacy due to numerous conditions and symptoms. This would constitute a major step for patients’ security. As mirtazapine is a very cheap drug, this would also contribute in limiting health care expenditures.

Besides limiting polypharmacy, several symptoms have very limited therapeutic options currently available. This is the case for cancer anorexia. Few drugs, including megestrol acetate and corticoids, have been proven to be effective in improving appetite. However, they have important side effects, limiting their use in the overall advanced cancer population. As mirtazapine is expected to be well tolerated, any effectiveness sin improving appetite might open an interesting therapeutic way for anorexia in advanced cancer.

For breathlessness treatment, no drug is currently licensed in the European Union. Morphine has been evaluated many times, but its effectiveness is still under debate. King’s College London recently launched an ambitious project aiming to assess the effectiveness of mirtazapine in alleviating breathlessness in chronic lung disease [32]. Results from this wide UK randomized control trial and form our study could motivate considering licensing mirtazapine in treating this symptom.

Additionally, mirtazapine is available in many countries under the shape of orodispersible tablets. This galenic form ensures its use for most cancer patients, including those who have swallowing disorders, intestinal obstruction or any other contraindication for oral intakes, while limiting venous access when not necessary.

Beyond the potential benefits to the patients and public health this study will inform further researches by providing information on advanced cancer patients’ quality of life, their experience of polysymptomatology and polypharmacy. This will also provide information on further researches on mirtazapine. Finally, feedback from this mixed-method randomized-controlled trial will provide useful information to subjective and objective aspects of research in palliative care patients’ quality of life, and thereafter suggests future methodological improvements.

### Limitations

For practical reasons, the current trial will be an open-trial. A recent modification in grading the evidence does not inevitably consider open-labeled trials as subject to important biases. Despite this new grading system, a blinded trial would have probably provided better evidence. To avoid biases, analyses, including qualitative analyses, will be performed by blinded researchers [33]. In this purpose, the researcher that will transcribe the semi-structured interviews verbatim will remove any identifiable data in the verbatim and blind them for randomization arm. This researcher will not take part in qualitative analyses.

## Data Availability

The current protocol is labeled by the number NCT04763135 on ClinicalTrials.gov. The current protocol is labeled by the number 2020–002,994-90 on the European Union Drug Regulating Authorities Clinical Trials Database. Other data are available upon reasonable request to the corresponding author.

## Abbreviations

ASEC: Antidepressant Side-Effect Checklist
CCS: Canadian Cancer Society
CTCAE V4.0 / CTACAE: Common Terminology Criteria for Adverse Events Fourth Version
ECG: Electrocardiogram
EORTC QLQ-C30 / QLQ-C30: European Organisation for Research and Treatment of Cancer Quality of Life Questionnaire 30
ESAS 12F: Edmonton Symptom Assessment Scale 12 French
ESMO: European Society for Medical Oncology
GHS: Global Health Status
HRQoL: Health-Related Quality of life
MADRS: Montgomery-Åsberg Depression Rating Scale
HADS-D: Hospital Anxiety and Depression Scale—Depression
MARS: Medication Adherence Report Scale
